# A Three-Year Study on the Nutritional Composition and Occurrence of Mycotoxins of Corn Varieties with Different Transgenic Events Focusing on Poultry Nutrition

**DOI:** 10.3390/vetsci11020097

**Published:** 2024-02-19

**Authors:** Juliano Kobs Vidal, Cristina Tonial Simões, Adriano Olnei Mallmann, Denize Tyska, Helder Victor Pereira, Carlos Augusto Mallmann

**Affiliations:** 1 Department of Preventive Veterinary Medicine, Federal University of Santa Maria, Santa Maria 97105-900, RS, Brazil; juliano.k.vidal@gmail.com (J.K.V.); crists02@gmail.com (C.T.S.); 2 Pegasus Science, Santa Maria 97105-030, RS, Brazil; adriano.mallmann@pegasusscience.com.br (A.O.M.); denize.tyska@pegasusscience.com.br (D.T.); 3 Cooperativa Agroindustrial Consolata, Cafelândia 85415-000, PR, Brazil; helder.pereira@copacol.com.br

**Keywords:** transgenic corn, poultry nutrition, mycotoxins, digestible amino acids, agronomic traits

## Abstract

**Simple Summary:**

Annually, various corn hybrids are introduced to the market for cultivation. Companies engaged in developing these technologies aim to enhance genetic traits, with a focus on creating productive hybrids capable of addressing the challenges of agriculture. The global corn market predominantly concerns livestock feed production, particularly for poultry farming, which seeks nutrient-rich raw materials with high digestibility for broilers. Nevertheless, when comparing the diverse transgenic technologies of corn, even when cultivated under the same conditions, significant differences were observed. Surprisingly, in the present study, the most productive corn transgenic technology in the field exhibited increased contamination by mycotoxins and a lower content of some important nutrients for poultry. This outcome highlights the critical need for a comprehensive assessment of the implications of transgenic technologies for nutritional composition and agricultural product safety, especially when intended for animal feed. Consequently, we concluded that the integration of nutritional considerations into the genetic improvement of transgenic corn, along with detailed information about resistance to *Fusarium*, holds great significance and may yield positive outcomes in the future. This approach ensures the production of nutritionally balanced, mycotoxin-safe, and economically viable livestock feed.

**Abstract:**

Corn is one of the most produced cereals in the world and plays a major role in poultry nutrition. As there is limited scientific information regarding the impact of transgenic technology on the quality and nutrient composition of the grains, this study investigated the effect of three major transgenic corn varieties—VT PRO3^®^, PowerCore^®^ ULTRA, and Agrisure^®^ Viptera 3—on the field traits, nutrient composition, and mycotoxin contamination of corn grains cultivated in southern Brazil during three consecutive harvests. VT PRO3^®^, while demonstrating superior crop yield, showed susceptibility to mycotoxins, particularly fumonisins. In contrast, PowerCore^®^ ULTRA, with the lowest yield, consistently exhibited lower levels of fumonisins. VT PRO3^®^ had higher AME_n_ than the other varieties, while PowerCore^®^ ULTRA had the highest total and digestible amino acid contents over the three years. The study’s comprehensive analysis reveals the distinct impact of transgenic corn technologies on both productivity and nutritional levels. Balancing the crops yield, mycotoxin resistance, and nutritional content of corn is crucial to meet the demands of the poultry feed industry. Such insights are essential for decision-making, ensuring sustainability and efficiency in agricultural production as well as meeting the demands of the poultry industry.

## 1. Introduction

Corn ranks among the most globally cultivated cereals, with the production of more than 1200 million tons in the 2022/2023 harvest, mainly concentrated in the United States, China, and Brazil [1]. In Brazil, corn is the second most produced grain, following soybeans. For the 2023/2024 harvest, the estimated production in Brazil will exceed 118 million tons [2]. In the Brazilian market, the diversity of corn cultivars is substantial, whereby 98, 259, and 98 different cultivars were available for commercialization in the 2020, 2021, and 2022 harvests, respectively [3,4,5]. Remarkably, the presence of transgenic cultivars has increased, accounting for 76%, 71%, and 95% of the total for the corresponding harvests. Among the transgenic cultivars, VT PRO3^®^, PowerCore^®^ ULTRA, and Agrisure^®^ Viptera 3 have emerged as the primary transgenic events, together representing 46% of available technologies in 2020, 64% in 2021 and 56% in 2022 [3,4,5].

Over the last decade, corn has consistently constituted approximately 80% of the total volume in the global trade of cereal grains, which includes corn, sorghum, barley, and oats [1]. The relevance of corn for the feed industry is evident, as around 70% of the corn marketed in Brazil is intended for animal nutrition [5], with it being mainly consumed by the poultry and swine industries. Corn is considered a high nutritional value ingredient, which contributes approximately 65% of the metabolizable energy and 20% of the protein to a broiler’s diet [6]. In addition to its recognized nutritional value, corn is also known as a natural source of carotenoids and xanthophyll [7], important pigments for the poultry industry, with them being deposited into the poultry skin and egg yolk. Despite being marketed as a commodity, there is substantial variability in its nutritional characteristics caused by several factors such as seed genetics, endosperm texture, cultivation location, climatic conditions, post-harvest management, and storage [8,9]. The current scenario emphasizes the importance of understanding the nutritional nuances of corn and the main factors involved.

The genetic improvement of corn has mainly been targeting high-productivity cultivars, with resistance to root lodging, and specific pathogens. However, there is an information gap concerning resistance to *Fusarium* in the corn hybrids currently marketed. In the 2020, 2021, and 2022 harvests, 82%, 99%, and 96% of hybrids, respectively, lacked information on *Fusarium* resistance [3,4,5]. Various strains of filamentous fungi, including *Aspergillus*, *Fusarium*, and *Penicillium*, are prevalent contaminants in corn crops. These fungal groups produce mycotoxins, secondary metabolites associated with well-documented toxic, mutagenic, and carcinogenic effects, leading to significant impacts on animal health as well as economic losses [10,11,12]. South American countries typically have high occurrences of fumonisins in corn, mycotoxins produced by *Fusarium* fungi that were found to contaminate more than 90% of the corn samples evaluated in different studies [13,14,15].

The management of mycotoxicological contamination in the animal feed chain involves several strategies. Reducing the moisture content of grains before storage as well as controlling and monitoring humidity and temperature during storage can significantly reduce the production of mycotoxins [11]. Other common strategies are the utilization of organic acids to reduce fungal contamination in grains and feeds [12], as well as the inclusion of antimycotoxin additives in the diet, which are capable of reducing the absorption of mycotoxins by the animals’ gastrointestinal tract [16].

Since there are no certified corn transgenic technologies regarding nutritional quality and susceptibility to mycotoxin-producing fungi, this study aims to fill part of this gap by analyzing the differences among the major transgenic events available for cultivation in the Southern region of Brazil (VT PRO3^®^, PowerCore^®^ ULTRA, and Agrisure^®^ Viptera 3) with respect to agronomic traits, nutrient composition, and contamination by mycotoxins.

## 2. Materials and Methods

### 2.1. Classification of Corn Types

Different commercial corn hybrids of each transgenic technology were chosen based on their commercialization rate in the region of the study and grouped into three categories of transgenic events: VT PRO3^®^, PowerCore^®^ ULTRA, and Agrisure^®^ Viptera 3. A total of 87 corn samples were evaluated (VT PRO3^®^ = 30, PowerCore^®^ ULTRA = 42, Agrisure^®^ Viptera 3 = 15) in 2020, 80 corn samples were evaluated (VT PRO3^®^ = 44, PowerCore Ultra = 36) in 2021, and 48 corn samples were evaluated (VT PRO3^®^ = 28, PowerCore^®^ ULTRA = 20) in 2022. The Agrisure^®^ Viptera 3 technology did not have commercial representation in 2021 and 2022 and, therefore, was not evaluated in these two years. To maintain confidentiality, the designations of the corn hybrids were kept undisclosed.

### 2.2. Field Experiments

The samples of corn from the three years of the study were obtained from experimental field plots cultivated at the Agricultural Research Center of the Cooperativa Agroindustrial Consolata (COPACOL), located in the state of Paraná, Brazil (24°37′01.800″ S, 53°18′02.000″ W, 580 m altitude). The region features dystrophic red latosol as its predominant soil type. Fertilization of crops was guided by chemical analyses and the nutritional requirements of the soil. Meteorological information such as precipitation, air temperature (°C), and relative humidity (%) was obtained over the three years of cultivation by a weather station positioned 50 m away from the experimental plots. The recorded data corresponded to the months when corn cultivation took place each year.

Corn crops from the three years were cultivated in a consolidated no-till system, under the same soil type. The field trials were arranged in a randomized block design, with each corn hybrid being a block with three replications by corn hybrid in 2020 and four replications by corn hybrid in both 2021 and 2022. In 2020, cultivation took place in the second half of January, with experimental plots containing four corn rows spaced 0.68 m apart and extending 14 m in length. In 2021 and 2022, cultivation occurred in the initial half of February, and the experimental plots contained four corn rows spaced 0.70 m apart and extending 6 m in length.

Treatment of seeds was implemented consistently during the three-year period, employing 300 mL/ha of thiodicarb + imidacloprid (Cropstar, Bayer, São Paulo, SP, Brazil). Insecticides and herbicides were administered following the guidelines provided by the manufacturers. These included 250 mL/ha of thiamethoxam + lambda-cyhalothrin (EngeoPleno, Syngenta, São Paulo, SP, Brazil), 2 L/ha of mesotrione + atrazine (Calaris, Syngenta, São Paulo, SP, Brazil), 150 mL/ha of lambda-cyhalothrin + chlorantraniliprole (Ampligo, Syngenta, São Paulo, SP, Brazil), and 100 mL/ha of spinetoram (Exalt, Corteva, Barueri, SP, Brazil), applied at the vegetative growth stages V1, V2, V3, and V5, respectively. Harvesting took place in the second half of June 2020 and the first half of July 2021 and 2022. The central two rows of each plot were harvested utilizing a Wintersteiger^®^ experimental plot harvester.

The mass of grains and moisture content were automatically determined by the Easy Harvest weighing system (Wintersteiger, Ried im Innkreis, OÖ, Austria), with the assistance of data collection systems Grain Gage^®^ (HarvestMaster, Logan, UT, USA) coupled with the harvesting system. The crop yield of the plots was calculated in kg/ha and adjusted for 13% moisture. Damaged grains were classified according to MAPA recommendations [17] and the percentage was obtained by the equation: [weight of damaged grains (g)/weight of the sample (g)] × 100.

### 2.3. Quantification of Mycotoxins via High-Performance Liquid Chromatography Coupled with Tandem Mass Spectrometry (HPLC-MS/MS)

After harvest, the samples were dried in a forced-air oven. A temperature of 55 °C was maintained for a period of 12 h, aiming to reduce the moisture content of the samples to approximately 13%. The dried samples (±1 kg) were sent to the Laboratory of Mycotoxicological Analysis at the Federal University of Santa Maria, Brazil. The samples were ground at 1 mm in an ultracentrifugal mill, model ZM 200(RETSCH^®^, Haan, NRW, Germany), homogenized, and subsequently analyzed for the presence and concentration of mycotoxins.

#### 2.3.1. Chemical Reagents

Analytical standards for aflatoxins (AF), fumonisins (FUM), deoxynivalenol (DON), and zearalenone (ZEA) were obtained from Sigma Aldrich (St. Louis, MO, USA). Acetonitrile, ammonium acetate, formic acid, and methanol (HPLC grade) were acquired from JT Baker (Center Valley, PA, USA). Ultra-pure water was obtained from a Milli-Q Advantage A10 Water Purification System (Merck KGaA, Darmstadt, HE, Germany).

#### 2.3.2. Aflatoxins (AFB_1_, AFB_2_, AFG_1_, and AFG_2_)

The method described by Mallmann et al. [18] was conducted for AF analyses. A sample of 5 g was mixed with 20 mL of an acetonitrile:water solution (84:16, *v*/*v*) and shaken for 60 min on a shaking table. The resulting extract was centrifuged (Eppendorf 5804R) at 2500 rpm, 20 °C, for 5 min. Then, 60 μL was diluted in 840 μL of a methanol:water solution (1:1, *v*/*v*) in a vial, and 20 μL of the obtained solution was then injected into an HPLC Infinity Series 1200 instrument (Agilent, Palo Alto, CA, USA) coupled to a 5500 QTRAP mass spectrometer (Applied Biosystems, Foster City, CA, USA). This system was equipped with an electrospray ionization (ESI) source in positive mode. Chromatographic separation was carried out at 30 °C using an Eclipse XDB-C8 column (4.6 × 150 mm, particle size 5 μm) (Agilent). The mobile phases consisted of water:ammonium acetate (99:1, *v*/*v*) and methanol:water:ammonium acetate (95:4:1, *v*/*v*/*v*).

#### 2.3.3. Deoxynivalenol and Zearalenone

For the assessment of DON and ZEA, the method described by Berthiller et al. [19] was applied. In this procedure, a 3 g sample was combined with 24 mL of a methanol:water mixture (70:30, *v*/*v*) and stirred for 20 min on an orbital shaker. Following this, the resultant extract was submitted to centrifugation at 2500 rpm, 20 °C, for 5 min. Subsequently, 40 μL of the centrifuged extract was diluted in 960 μL of a methanol:water:ammonium acetate solution (90:9:1, *v*/*v*/*v*) in a vial. A 10 μL aliquot of this solution was introduced into an HPLC Infinity Series 1200 instrument (Agilent Technologies, Santa Clara, CA, USA) coupled to a 5500 QTRAP mass spectrometer (Applied Biosystems, Waltham, MA, USA), featuring an ESI source in positive mode. Chromatographic separation was carried out at 40 °C using a Zorbax SB-C18 column (4.6 × 150 mm, particle diameter of 5 μm). The mobile phases were methanol:water:ammonium acetate (90:9:1, *v*/*v*/*v*) and water:ammonium acetate (90:10, *v*/*v*).

#### 2.3.4. Fumonisins (FB_1_ and FB_2_)

The analyses of FUM were performed according to the method of Mallmann et al. [18]. A 3 g sample was added to a Falcon tube with 15 mL of a solution with acetonitrile and water in a 1:1 ratio (*v*/*v*). The tube was shaken for 20 min using an orbital shaker. Afterward, the resultant mixture was centrifuged at 2500 rpm, 20 °C, for 5 min, and 20 μL was diluted in 980 μL of a solution containing acetonitrile, water, and formic acid in a 50:40:10 ratio (*v*/*v*/*v*). Then, 10 μL of the obtained solution was introduced into an HPLC Infinity Series 1200 apparatus (Agilent), connected to an API 5000 mass spectrometer (Applied Biosystems) featuring an ESI source in positive mode. Chromatographic separation was conducted at 40 °C using an Eclipse XDB-C8 column (4.6 × 150 mm, particle diameter of 5 μm). The mobile phases comprised acetonitrile and formic acid (95:5, *v*/*v*) and water and formic acid (95:5, *v*/*v*).

#### 2.3.5. Parameters of Method Performance

The quantification limits (LOQ) and detection limits (LOD) of each mycotoxin were determined by evaluating the signal-to-noise ratio (LOQ = 10/1; LOD = 3/1). The recovery rate (%) of each method (AF, FUM, DON, and ZEA) was based on the mean concentration obtained from corn-fortified samples with three different levels of the target analyte (mycotoxin) with seven replicates each. The linearity of the analytical curves from each mycotoxin was examined by utilizing the coefficient of determination (R^2^), which was computed following triplicate injections of the analytical curves at seven distinct concentration levels.

### 2.4. Near-Infrared Spectroscopy Nutritional Predictions

For predictions using near-infrared spectroscopy (NIRS), samples were milled at 0.5 mm in an ultra-centrifugal mill, placed in plastic bags, and left for 15 min to reach room temperature (between 18 °C and 22 °C) and humidity (between 40% and 60%). Subsequently, manual homogenization of each sample was performed for two minutes using circular movements. Nutritional predictions were performed by reading the spectra of the samples in a Bruker^®^ instrument, model Tango-R, with a wavelength range of 3952–11,536 cm^−1^, using the calibration curves from the AMINONRG^®^ and AMINONir^®^ programs (Evonik Nutrition & Care GmbH, Hanau, Germany). The following variables were predicted: dry matter (DM) (%), crude protein (CP) (%), ether extract (EE) (%), ash (%), total P (%), phytic P (%), total and digestible (dig.) amino acids (AA, %), and apparent metabolizable energy (AME_n_) (kcal/kg) for poultry. For study and comparison purposes, all values were adjusted to an 87% DM basis.

### 2.5. Statistical Analysis

The statistical analysis was conducted using SAS software, version 9.4, 2015 (SAS Institute, Cary, NC, USA). The normality of the data was tested through the use of the Shapiro–Wilk test prior to other analyses. The contamination data of all mycotoxins from the three years were transformed by log_10_(x + 1). Data were subjected to ANOVA using the GLIMMIX procedure. Means of mycotoxin contamination, field data, and nutritional variables from different transgenic technologies were compared using Tukey’s test. Significance was accepted at *p* < 0.05. The following statistical model was utilized:

У*ijk* = µ + τ*i* + β*j*+ ε*ijk*

where У*ijk* represents the observed response of the *i*-th transgenic technology in the *j*-th commercial hybrid and *k*-th replicate; µ is the overall mean; τ*i* is the fixed effect of the *i*-th transgenic technology; β*j* is the random effect of the *j*-th commercial hybrid; and ε*ijk* is the residual error.

## 3. Results

### 3.1. Meteorological Data

**Figure 1:**
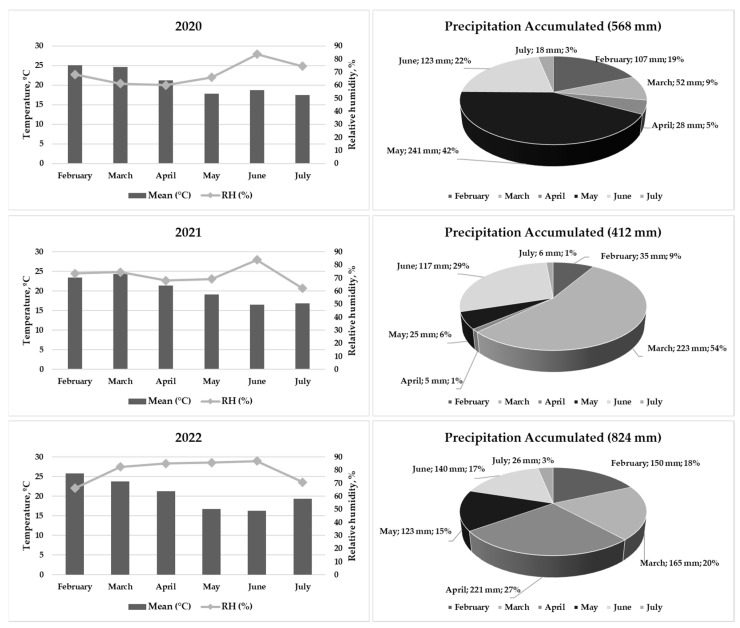
Climatic conditions during the cultivation of different transgenic technologies of corn in 2020, 2021, and 2022.

The analysis of meteorological data in 2020, 2021, and 2022 is represented in Figure 1. Daily average temperature (°C) and relative humidity (%) are presented on a monthly basis, while precipitation is expressed in cumulative millimeters (mm) per month.

The annual average temperature indicated overall stability among the years (21 °C in 2020, 20 °C in 2021, and 21 °C in 2022). In 2020, the monthly maximum and minimum averages were 25.1 and 17.4 °C, observed in February and July, respectively. In 2021, March exhibited the highest monthly average at 24.3 °C, whereas a minimum average of 16.5 °C was measured in June. For 2022, February had the highest monthly average at 25.9 °C, and the minimum average of 16.2 °C was measured in June.

In 2020, the average relative humidity was 69%, increasing to 72% in 2021 and reaching a maximum of 79% in 2022. The maximum and minimum averages for the periods were, respectively: 84% in June and 60% in April 2020; 84% in June and 62% in July 2021; and 87% in June and 66% in February 2022. Regarding accumulated precipitation, there was notable variation among the years, with 568 mm in 2020, 412 mm in 2021, and 824 mm in 2022. Monthly distribution also varied, as shown in the circle charts of Figure 1.

### 3.2. Damaged Grains, Crop Yield, and Mycotoxin Contamination

The R^2^ of the analytical curves for mycotoxin analyses presented values greater than 0.99. The LOD and LOQ (in μg/kg) for the evaluated mycotoxins were, respectively: 0.4 and 1 for AFB_1_; 0.6 and 1 for AFB_2_, AFG_1_, and AFG_2_; 10 and 125 for FB_1_; 20 and 125 for FB_2_; 50 and 200 for DON; and 3 and 20 for ZEA. The results for the damaged grains, crop yield, and mycotoxin concentration for different corn transgenic events from 2020, 2021, and 2022 are presented in Table 1.

In 2020, a significant difference in crop yield was observed among the three technologies, with VT PRO3^®^ exhibiting a higher yield (9029 kg/ha) compared to PowerCore^®^ ULTRA (8591 kg/ha) (*p* = 0.0411). Agrisure^®^ Viptera 3 presented intermediate productivity results (8767 kg/ha). Additionally, the occurrence of damaged grains was not different among corn transgenic events (*p* = 0.6283). The means of AF were not different among the transgenic technologies (*p* > 0.05) whereas DON and ZEA did not occur (<LOQ). However, total FUM (FB_1_ + FB_2_) was significantly higher in VT PRO3^®^ (1180 µg/kg) compared to PowerCore^®^ ULTRA (280.8 µg/kg) and Agrisure^®^ Viptera 3 (8.33 µg/kg) (*p* = 0.0001).

In 2021, an increase in the percentage of damaged grains was observed in the VT PRO3^®^ technology (1.66%) compared to PowerCore^®^ ULTRA (0.75%) (*p* = 0.0005). Similarly to the previous year, crop yield was higher in VT PRO3^®^ (5085 kg/ha) than in PowerCore^®^ ULTRA (4166 kg/ha) (*p* = 0.0002). Regarding mycotoxins, total FUM levels were again higher in VT PRO3^®^ (1657 µg/kg) compared to PowerCore^®^ ULTRA (414.0 µg/kg) (*p* = 0.0008), with a difference greater than 1000 µg/kg. The means of AF, DON, and ZEA were not different between the two corn transgenic technologies (*p* > 0.05).

In 2022, crop yield differences persisted, with VT PRO3^®^ presenting higher performance (9411 kg/ha) compared to PowerCore^®^ ULTRA (8806 kg/ha) (*p* = 0.0045). Additionally, a difference was observed in the concentration of DON, with it being higher in the PowerCore^®^ ULTRA technology (481.0 µg/kg) compared to the VT PRO3^®^ (138.8 µg/kg) (*p* = 0.0147). Regarding total FUM, consistent with the previous years’ results, VT PRO3^®^ (2566 µg/kg) presented higher means than PowerCore^®^ ULTRA (990.6 µg/kg) (*p* = 0.0127), representing a difference greater than 1500 µg/kg.

### 3.3. Proximal Composition and Phosphorus Values

The nutritional composition results for the different transgenic events of corn in the 2020, 2021, and 2022 crops are presented in Table 2. In 2020, although CP showed only a slight tendency among the technologies (*p* = 0.0603), CF and EE were statistically different (*p* = 0.0104 and *p* = 0.0001, respectively). Crude fiber and EE were higher in the VT PRO3^®^ technology than in PowerCore^®^ ULTRA and Agrisure^®^ Viptera 3. Other components, such as ash and P, were not different among the corn technologies (*p* > 0.05).

In 2021, there were significant variations in the concentrations of CP, ash, and EE between the corn technologies. Crude protein was higher in PowerCore^®^ ULTRA (10.02%) compared to VT PRO3^®^ (9.29%) (*p* = 0.0001). Ether extract was higher in VT PRO3^®^ (3.75%) compared to PowerCore^®^ ULTRA (3.58%) (*p* = 0.0113). The technologies also differed in their ash values (*p* = 0.0001), with VT PRO3^®^ showing the lowest content. Furthermore, PowerCore^®^ ULTRA presented higher levels of total (*p* = 0.0480) and phytic (*p* = 0.0483) P compared to VT PRO3^®^.

Differences in nutritional characteristics between corn transgenic technologies were also observed in 2022, with this being consistent with the results observed in the previous years. PowerCore^®^ ULTRA had a higher concentration of CP and ash and higher *p*-values than VTPRO3 (*p* < 0.05), whereas VT PRO3^®^ had the highest concentration of EE (*p* = 0.0001).

### 3.4. Amino Acids and Metabolizable Energy for Poultry

In 2020, corn transgenic events exhibited significant differences in certain total and dig. AA and in the AME_n_, as observed in Table 3. Total and dig. Ile, Leu, and Phe were higher in PowerCore^®^ ULTRA compared to VT PRO3^®^ and Agrisure^®^ Viptera 3 (*p* = 0.0338 and *p* = 0.0376; *p* = 0.0252 and *p* = 0.0255; and *p* = 0.0269 and *p* = 0.0193, respectively). Additionally, AME_n_ differed significantly among technologies, being higher in VT PRO3^®^ than in PowerCore^®^ ULTRA and Agrisure^®^ Viptera 3 (*p* = 0.0007). In 2021, all the total and dig. AA was significantly different between the corn technologies (*p* < 0.05), with higher concentrations in PowerCore^®^ ULTRA compared to VT PRO3^®^. In addition, AME_n_ was higher in VT PRO3^®^ compared to PowerCore^®^ ULTRA (*p* = 0.0014). In 2022, the PowerCore^®^ ULTRA technology had a higher concentration of most of the total and dig. AA than VT PRO3^®^ (*p* < 0.05), with the exception of total Lys (*p* = 0.1480) and dig. Trp (*p* = 0.0909). Furthermore, AME_n_ was significantly higher in VT PRO3^®^ compared to PowerCore^®^ ULTRA (*p* = 0.0001).

## 4. Discussion

The maximization of productivity per hectare in corn cultivation assumes significant relevance, given the evolution of human and animal nutrition, as well as the concern for environmental preservation [20]. Efficiency in the utilization of cultivated land plays a crucial role in meeting global food needs [21]. When it comes to corn intended for poultry feed, it is therefore important to consider materials with high safety, nutritional concentration, and nutrient digestibility [22].

Agricultural production is intrinsically dependent on climatic conditions. Climatic fluctuation has negative impacts on crop development, grain yield, and quality, influencing processes such as vegetative development, flowering, and grain maturation, as well as the incidence of pests and diseases [8]. The analysis of meteorological data collected in the experimental field over the three years of the present study enabled the identification of variability among the years and the discussion of the potential impact of these conditions on crop yield and grain quality. It has already been demonstrated that the meteorological variables from different years exert some influence on the nutritional and mycotoxicological composition of different corn hybrids [23,24]. Data from 2020, 2021, and 2022 reveal challenging climatic conditions for corn cultivation in the region where the present study was conducted.

Temperature is crucial for the corn cycle and should range between 24 °C and 30 °C from emergence to the flowering period. A daily average temperature of 21 °C is optimal for the highest grain yield, according to a study by EMBRAPA [25]. Despite the stability of the annual mean temperature over the three years of study, monthly variations were recorded, with maximum temperatures exceeding 25 °C in February and March and minimums below 17 °C in June and July. The optimal relative humidity range for corn cultivation is between 60% and 80% [26]. This variable plays a crucial role in plant transpiration, soil water availability, and the occurrence of fungal diseases. Our data indicated that the average relative humidity in the study region was within the expected range over the three years analyzed (69% in 2020, 72% in 2021, and 79% in 2022). However, values above 80% in June for all three years may have compromised crop health and grain quality and favored some mycotoxins’ occurrence. Notable variations in accumulated precipitation were observed over the three years (568 mm in 2020, 412 mm in 2021, and 824 mm in 2022), potentially impacting corn production, as well as fungal development favored by high humidity levels.

Overall, fluctuations in temperature, high relative humidity, and irregular precipitation were observed and are possibly related to the increase in FUM concentration from 2020 to 2022 as well as the higher levels of DON and ZEA observed in 2022, which could be explained by the higher precipitation observed in this last year. In 2020 and 2021, the lower temperature variation, coupled with humidity below 80%, except in June of both years, may have alleviated fungal stress, consequently leading to values below the LOQ for ZEA and DON in 2020 and low concentrations of these mycotoxins in 2021. Additionally, the temperature variation between April and May of 2022, coupled with an average humidity close to 90%, may have served as a stress factor for *Fusarium* fungi, triggering the production of FUM, DON, and ZEA in that year.

Data from the present study demonstrated that distinct transgenic technologies applied to corn influenced crop yield, the incidence of damaged grains, and the concentration of mycotoxins during the 2020, 2021, and 2022 harvests. Notably, the VT PRO3^®^ technology demonstrated superior yield in all years of the study, possibly related to the three Bt genes of this technology (Cry1A.105, Cry2Ab2, and Cry3Bb1) [27], which together promote resistance to insect pests (*Spodoptera frugiperda*, *Diatraea saccharalis*, *Helicoverpa zea*, *Elasmopalpus lignosellus*, and *Diabrotica speciosa*), in addition to herbicide tolerance (glyphosate) [5]. However, a higher concentration of fumonisins, mycotoxins mainly produced by *Fusarium verticillioides and F. proliferatum*, was observed. This result may indicate a higher susceptibility of VT PRO3^®^ to infection by fumonisins-producing fungi. In contrast to VT PRO3^®^, the PowerCore^®^ ULTRA technology exhibited a lower yield but a low concentration of total fumonisins. This technology carries four Bt genes (Cry1F, Cry1A.105, Cry2Ab2, and Vip3Aa20) [28], which provide resistance to different insect pests (*S. frugiperda*, *D. saccharalis*, *Helicoverpa armigera*, *H. zea*, *Elasmopalpus lignosellus*, *Agrotis ipsilon*, *S. eridania*, and *S. cosmioides*), in addition to herbicide tolerance (glyphosate) [5].

In 2022, PowerCore^®^ ULTRA had a high concentration of DON, a type B trichothecene mainly produced by *Fusarium graminearum* and *F. culmorum*. This result suggests a possible higher susceptibility of PowerCore^®^ ULTRA to DON-producing species. Alternatively, Agrisure^®^ Viptera 3 presented intermediate results among the three transgenic events, with comparable crop yield to PowerCore^®^ ULTRA and low concentration of total fumonisins. Agrisure^®^ Viptera 3 has three Bt genes (Cry1Ab, Vip3Aa20, and Cp4-EPSPS) [29], conferring resistance to various insect pests (*S. frugiperda*, *D. saccharalis*, *H. zea*, *E. lignosellus*, and *A. ipsilon*) and herbicide tolerance (glyphosate) [5]. In 2020, this transgenic technology presented, numerically, the lowest concentration of total fumonisins among the evaluated events. This finding suggests a possible resistance against fumonisins-producing *Fusarium* species.

The mycotoxicological results from the present study emphasize the imperative need to monitor the factors related to FUM production in different transgenic corn. This group of mycotoxins has a significant prevalence in Brazilian and South American corn, as evidenced by various prior surveys [13,30]. Poultry exposed to fumonisins typically manifest mild to moderate toxicity, characterized by notable changes such as liver pathology, increased intestinal permeability, and decreased growth performance [31,32].

The insertion of genes in transgenic events of corn, intended to improve agronomic traits such as resistance to herbicides, insects, abiotic stresses, and diseases, may affect its nutritional composition compared to conventional corn [33]. The results regarding concentrations of CP, CF, ash, and EE in the present study revealed differences in nutritional characteristics among transgenic technologies within each year and a consistent pattern over the years, especially regarding CP and EE. In 2020, despite the numerical difference, CP was the same among the technologies, while CF and EE had higher concentrations in the VT PRO3^®^ technology. In 2021 and 2022, all components were significantly different between the transgenic events, except for CF. Consistent results on nutrient compositions were observed over the years in the present study; PowerCore^®^ ULTRA presented higher levels of CP, ash, and P values compared to VT PRO3^®^ in 2021 and 2022 whereas VT PRO3^®^ had the highest EE content during the three years. Such differences can be attributed to environmental and genetic factors and their interactions, influencing the metabolism and composition of corn grains [34]. Piovesan et al. [35] and Vieira et al. [36] observed that protein is influenced by the corn hybrid, production year, cultivation region, and meteorological data. Variations in the concentrations of CP, CF, ash, and ether extract among corn technologies highlight the need for constant monitoring of corn nutritional composition since different corn varieties are consumed at poultry feed mills on a daily basis. In addition, results from the present study demonstrate that the choice of one transgenic technology can thus directly influence the nutritional density of the final feed.

According to Cowieson [6], corn can represent around 65% and 20% of the energy and protein supplies in broiler starter diets, respectively. Therefore, any difference observed in the composition of this ingredient impacts the cost of feed formulation. The results of total and digestible amino acids as well as metabolizable energy indicated remarkable variations among the transgenic technologies. These differences can be attributed not only to the genetic characteristics but also tissue structure of corn grains [9]. In 2020, a significant difference in the dig. Ile, Leu, and Phe were observed among corn technologies, with PowerCore^®^ ULTRA and Agrisure^®^ Viptera 3 standing out with the highest levels. In 2021, reinforcing the data obtained in 2020, all dig. AA in the PowerCore^®^ ULTRA technology was higher compared to the VT PRO3^®^ technology. In 2022, only dig. Trp was not different between the transgenic events, while the other dig. AA had the highest concentration in PowerCore^®^ ULTRA, corroborating the findings of 2020 and 2021. Additionally, VT PRO3^®^ presented the highest levels of AME_n_ in the three years of the study. It is possible that the high AME_n_ levels in VT PRO3^®^ are related to the higher EE content in the grains of this transgenic technology, which was also observed during the three years of the present study.

## 5. Conclusions

The quality of corn is influenced by climatic, technological, and genetic factors. The transgenic technologies evaluated herein have played distinct roles, with VT PRO3^®^ standing out in crop yield but also showing potential susceptibility to fumonisin contamination. In spite of the lower crop yield, PowerCore^®^ ULTRA had lower concentrations of total fumonisins. The variation in nutritional characteristics among corn technologies over the years and notable differences in digestible amino acids and metabolizable energy highlight the importance of constant evaluations of corn nutritional composition to optimize the feed efficiency of poultry. The differences among corn technologies, both in productivity and nutritional levels, demonstrate the importance of incorporating the “nutritional content” bias in the selection and improvement process of corn hybrids/genetics.

In conclusion, selecting transgenic events for corn production intended for poultry feed formulation should be based not only on crop yield but also on the quality of grains, the presence of mycotoxins, and specific nutritional characteristics. This well-informed decision-making is essential to ensure sustainability and efficiency in agricultural production and to meet the demands of the poultry industry.

## Figures and Tables

**Table 1:** Damaged grains, crop yield, and mycotoxin concentration in the different transgenic technologies of corn: 2020, 2021, and 2022.

**2020**					
	**Transgenic Technology**				
**Item**	**VT PRO3^®^**	**PowerCore^®^** **ULTRA**	**Agrisure^®^** **Viptera 3**	**SEM**	***p*-Value**
Damaged grains, %	0.21	0.15	0.22	0.034	0.6283
Crop yield, kg/ha	9029 ^a^	8591 ^b^	8767 ^ab^	85.81	0.0411
Total aflatoxins ^1^ (µg/kg)	1.49	0.29	0.28	0.393	0.3518
Deoxynivalenol (µg/kg)	<LOQ ^3^	<LOQ	<LOQ		
Total fumonisins ^2^ (µg/kg)	1180 ^a^	280.8 ^b^	8.33 ^b^	94.22	0.0001
Zearalenone (µg/kg)	<LOQ	<LOQ	<LOQ		
**2021**					
	**Transgenic Technology**				
**Item**	**VT PRO3^®^**	**PowerCore^®^ ULTRA**		**SEM**	***p*-Value**
Damaged grains, %	1.66 ^a^	0.75 ^b^		0.134	0.0005
Crop yield, kg/ha	5085 ^a^	4166 ^b^		127.2	0.0002
Total aflatoxins (µg/kg)	0.256	0.194		0.054	0.5705
Deoxynivalenol (µg/kg)	21.75	19.00		7.634	0.8591
Total fumonisins (µg/kg)	1657 ^a^	414.0 ^b^		190.0	0.0008
Zearalenone (µg/kg)	11.80	1.56		2.728	0.0717
**2022**					
	**Transgenic Technology**				
**Item**	**VT PRO3^®^**	**PowerCore^®^ ULTRA**		**SEM**	***p*-Value**
Damaged grains, %	2.23	3.35		0.363	0.1304
Crop yield, kg/ha	9411 ^a^	8806 ^b^		107.8	0.0045
Total aflatoxins (µg/kg)	0.800	0.580		0.178	0.5491
Deoxynivalenol (µg/kg)	138.8 ^b^	481.0 ^a^		70.28	0.0147
Total fumonisins (µg/kg)	2566 ^a^	990.6 ^b^		317.2	0.0127
Zearalenone (µg/kg)	131.7	345.5		57.57	0.0766

^a–b^ Means with different superscript letters differ (*p* < 0.05) based on Tukey’s honestly significant difference test. ^1^ Sum of aflatoxins B_1_, B_2_, G_1_, and G_2_. ^2^ Sum of fumonisins B_1_ and B_2_. ^3^ LOQ, limit of quantification.

**Table 2:** Nutrient composition in the different transgenic technologies of corn: 2020, 2021, and 2022.

**2020**					
	**Transgenic Technology**				
**Variable**	**VT PRO3^®^**	**PowerCore^®^** **ULTRA**	**Agrisure^®^** **Viptera 3**	**SEM**	***p*-Value**
Crude protein, %	8.21	8.44	8.44	0.046	0.0603
Crude fiber, %	2.12 ^a^	2.03 ^b^	2.09 ^ab^	0.014	0.0104
Ash, %	1.14	1.15	1.13	0.006	0.3168
Ether extract, %	4.03 ^a^	3.77 ^b^	3.69 ^b^	0.030	0.0001
Total P, mg/kg	1965	1959	1963	13.11	0.9761
Phytic P, mg/kg	1474	1469	1472	9.83	0.9750
**2021**					
	**Transgenic Technology**				
**Variable**	**VT PRO3^®^**	**PowerCore^®^ ULTRA**		**SEM**	***p*-Value**
Crude protein, %	9.29 ^b^	10.02 ^a^		0.082	0.0001
Crude fiber, %	1.99	2.00		0.021	0.8422
Ash, %	1.19 ^b^	1.27 ^a^		0.008	0.0001
Ether extract, %	3.75 ^a^	3.58 ^b^		0.033	0.0113
Total P, mg/kg	2012 ^b^	2094 ^a^		15.70	0.0084
Phytic P, mg/kg	1509 ^b^	1571 ^a^		11.78	0.0084
**2022**					
	**Transgenic Technology**				
**Variable**	**VT PRO3^®^**	**PowerCore^®^ ULTRA**		**SEM**	***p*-Value**
Crude protein, %	7.95 ^b^	8.87 ^a^		0.093	0.0001
Crude fiber, %	1.96	1.91		0.022	0.3069
Ash, %	1.19 ^b^	1.23 ^a^		0.009	0.0326
Ether extract, %	3.70 ^a^	3.36 ^b^		0.038	0.0001
Total P, mg/kg	1970 ^b^	2045 ^a^		18.83	0.0480
Phytic P, mg/kg	1477 ^b^	1533 ^a^		14.14	0.0483

^a–b^ Means with different superscript letters differ (*p* < 0.05) based on Tukey’s honestly significant difference test.

**Table 3:** Total and digestible amino acids and metabolizable energy for poultry in different transgenic technologies of corn: 2020, 2021, and 2022.

**2020**					
	**Transgenic Technology**				
**Variable**	**VT PRO3^®^**	**PowerCore^®^** **ULTRA**	**Agrisure^®^** **Viptera 3**	**SEM**	***p*-Value**
Total Met + Cys, %	0.354	0.359	0.363	0.0015	0.0700
Dig ^1^. Met + Cys, %	0.326	0.331	0.334	0.0014	0.0915
Total Lys, %	0.231	0.230	0.231	0.0009	0.8598
Dig. Lys, %	0.210	0.210	0.211	0.0008	0.9845
Total Thr, %	0.291	0.298	0.299	0.0015	0.0954
Dig. Thr, %	0.259	0.265	0.266	0.0013	0.1082
Total Trp, %	0.061	0.061	0.062	0.0002	0.7187
Dig. Trp, %	0.051	0.050	0.050	0.0001	0.1465
Total Arg, %	0.379	0.383	0.385	0.0017	0.4068
Dig. Arg, %	0.338	0.341	0.342	0.0014	0.4358
Total Val, %	0.390	0.399	0.401	0.0020	0.0808
Dig. Val, %	0.371	0.380	0.378	0.0019	0.1122
Total Ile, %	0.281 ^b^	0.290 ^a^	0.291 ^a^	0.0018	0.0338
Dig. Ile, %	0.276 ^b^	0.285 ^a^	0.288 ^a^	0.0017	0.0376
Total Leu, %	1.021 ^b^	1.064 ^a^	1.070 ^a^	0.0079	0.0252
Dig. Leu, %	0.950 ^b^	0.990 ^a^	0.995 ^a^	0.0074	0.0255
Total His, %	0.241	0.246	0.246	0.0011	0.1089
Dig. His, %	0.233	0.238	0.239	0.0011	0.0677
Total Phe, %	0.386 ^b^	0.412 ^a^	0.414 ^a^	0.0029	0.0269
Dig. Phe, %	0.368 ^b^	0.383 ^a^	0.386 ^a^	0.0027	0.0193
AME_n_^2^, kcal/kg	3340 ^a^	3330 ^b^	3326 ^b^	1.4952	0.0007
**2021**					
	**Transgenic Technology**				
**Variable**	**VT PRO3^®^**	**PowerCore^®^ ULTRA**		**SEM**	***p*-Value**
Total Met + Cys, %	0.367 ^b^	0.384 ^a^		0.0029	0.0043
Dig. Met + Cys, %	0.334 ^b^	0.350 ^a^		0.0026	0.0025
Total Lys, %	0.242 ^b^	0.251 ^a^		0.0014	0.0030
Dig. Lys, %	0.213 ^b^	0.221 ^a^		0.0013	0.0020
Total Thr, %	0.321 ^b^	0.345 ^a^		0.0028	0.0001
Dig. Thr, %	0.277 ^b^	0.297 ^a^		0.0023	0.0001
Total Trp, %	0.064 ^b^	0.067 ^a^		0.0003	0.0005
Dig. Trp, %	0.053 ^b^	0.057 ^a^		0.0005	0.0016
Total Arg, %	0.406 ^b^	0.426 ^a^		0.0028	0.0004
Dig. Arg, %	0.361 ^b^	0.379 ^a^		0.0025	0.0004
Total Val, %	0.430 ^b^	0.462 ^a^		0.0036	0.0001
Dig. Val, %	0.400 ^b^	0.430 ^a^		0.0034	0.0001
Total Ile, %	0.319 ^b^	0.347 ^a^		0.0031	0.0001
Dig. Ile, %	0.307 ^b^	0.333 ^a^		0.0029	0.0001
Total Leu, %	1.169 ^b^	1.288 ^a^		0.0137	0.0001
Dig. Leu, %	1.076 ^b^	1.185 ^a^		0.0127	0.0001
Total His, %	0.256 ^b^	0.272 ^a^		0.0020	0.0002
Dig. His, %	0.244 ^b^	0.257 ^a^		0.0019	0.0005
Total Phe, %	0.453 ^b^	0.498 ^a^		0.0052	0.0001
Dig. Phe, %	0.417 ^b^	0.459 ^a^		0.0048	0.0001
AME_n_, kcal/kg	3328 ^a^	3317 ^b^		1.8655	0.0014
**2022**					
	**Transgenic Technology**				
**Variable**	**VT PRO3^®^**	**PowerCore^®^ ULTRA**		**SEM**	***p*-value**
Total Met + Cys, %	0.349 ^b^	0.367 ^a^		0.0038	0.0226
Dig. Met + Cys, %	0.310 ^b^	0.338 ^a^		0.0034	0.0001
Total Lys, %	0.240	0.248		0.0016	0.1480
Dig. Lys, %	0.209 ^b^	0.217 ^a^		0.0015	0.0071
Total Thr, %	0.285 ^b^	0.304 ^a^		0.0032	0.0014
Dig. Thr, %	0.241 ^b^	0.266 ^a^		0.0020	0.0001
Total Trp, %	0.060 ^b^	0.063 ^a^		0.0004	0.0117
Dig. Trp, %	0.050	0.051		0.0002	0.0909
Total Arg, %	0.371 ^b^	0.388 ^a^		0.0033	0.0089
Dig. Arg, %	0.325 ^b^	0.348 ^a^		0.0029	0.0001
Total Val, %	0.381 ^b^	0.407 ^a^		0.0041	0.0017
Dig. Val, %	0.347 ^b^	0.386 ^a^		0.0038	0.0001
Total Ile, %	0.278 ^b^	0.309 ^a^		0.0036	0.0014
Dig. Ile, %	0.260 ^b^	0.295 ^a^		0.0035	0.0001
Total Leu, %	0.983 ^b^	1.088 ^a^		0.0171	0.0017
Dig. Leu, %	0.873 ^b^	1.021 ^a^		0.0151	0.0001
Total His, %	0.239 ^b^	0.248 ^a^		0.0025	0.0012
Dig. His, %	0.220 ^b^	0.239 ^a^		0.0024	0.0001
Total Phe, %	0.388 ^b^	0.427 ^a^		0.0063	0.0017
Dig. Phe, %	0.356 ^b^	0.401 ^a^		0.0060	0.0001
AME_n_, kcal/kg	3329 ^a^	3311 ^b^		1.9347	0.0001

^a–b^ Means with different superscript letters differ (*p* < 0.05) based on Tukey’s honestly significant difference test. ^1^ Ileal digestible amino acids for poultry (predicted using AMINONIR^®^ calibration curves). ^2^ AME_n_ = apparent metabolizable energy for poultry (predicted using AMINONRG^®^ calibration curves).

## Data Availability

Data related to this research are available within the article.
